# We need to talk about migrant minors’ health

**DOI:** 10.1016/j.lana.2021.100125

**Published:** 2021-11-11

**Authors:** The Lancet Regional Health - Americas

According to the 2020 UN High Commissioner for Refugees (UNHCR) global report, 4•9 million Venezuelan refugees and 550 000 migrants from El Salvador, Guatemala, and Honduras have been forcibly displaced, seeking international protection predominantly in the Americas or Caribbean. Approximately 24% of those displaced are minors travelling with their families or seeking to be reunited with them. Migration and forced displacement can be traumatic for all individuals; however, children and adolescents face additional physical and mental health risks. These risks can include, for example, deprivation, hunger, exploitation, extortion, and abuse, all of which have short-term and long-term implications to migrating minors’ health.

Mental health conditions—such as post-traumatic stress disorder (PTSD), depression, anxiety, substance use, internalising and externalising behaviour, social withdrawal, and stress—are frequent consequences faced by migrating minors. The 1989 UN Convention on the Rights of the Child (CRC) declared that the country of destination has a duty to provide protection and humanitarian assistance to any child seeking asylum. However, an increasing body of evidence exposes a different reality. Detention of minors in country border facilities, under the guise of protection, is widespread, and reports of unsuitable accommodations, scarce food and water supply, and denial of adequate medical care are not uncommon.

In 2019 alone, 76 020 minors, mainly from Honduras, Guatemala, and El Salvador, were detained at the USA–Mexico border. Children and adolescents flee their homes for a multitude of reasons: young boys hope to escape frequent harassment and threats from gang members, while girls and young LGBTQ+ people flee from physical and sexual abuse. In addition to the emotional burden of leaving their home and the harms of the journey, migrant children are also vulnerable to verbal, physical, and sexual abuse from border enforcement officials.

Trauma experienced at a young age can cause adverse effects on the developing brain, leading to enduring consequences. Forced migration may expose minors to recurring adverse events, such as physical, psychological, and sexual abuse; neglect; and household dysfunction. These can combine with the broader socioeconomic context—including other social determinants of health, structural racism, perceived discrimination, economic instability, and absence of a safety network—and lead to toxic stress. Physiological long-term effects of toxic stress include increased risk for infections and non-communicable diseases, while alcoholism, substance use, depression, and attempted suicide are among the reported mental consequences.

Unaccompanied minors are especially vulnerable to physical abuse, disease, and trauma, given the absence of a protective family unit. A higher prevalence of PTSD has been reported in unaccompanied than in accompanied minors. Nevertheless, unaccompanied minors can develop positive coping skills during migration, which can be built upon with the right tools and assistance to promote their wellbeing. Culturally sensitive, trauma-informed approaches to address mental health, applied in both short term and long term, are necessary to garner trust and enable suitable and long-lasting care for the minor.

Migrating children and adolescents are also vulnerable to poor physical health, including nutritional deficiencies (eg, malnourishment and dental caries), skin and gastrointestinal infections, latent tuberculosis, and hygiene-related parasitosis, such as schistosomiasis. Moreover, with little or no access to vaccination, many are exposed to vaccine-preventable diseases. Such harmful conditions can be easily addressed if host countries provide basic health-care services at every migration checkpoint, including sexual and reproductive health services for victims of sexual violence.

At the beginning of the COVID-19 pandemic, using an interpretation of Title 42, the Trump administration expelled or denied asylum to millions of migrants and refugees, including minors, under the premise of avoiding the spread of SARS-CoV-2 in the USA. Although the Biden administration has now made unaccompanied children exempt from Title 42, families detained at the border either return as a family to their home country or send their children and adolescents across the border unaccompanied. A record number of 33 000 unaccompanied children arrived at the USA–Mexico border during the first 3 months of 2021. Conversely, Colombia's government has granted protected status to 1•7 million migrants, allowing nearly 950 000 undocumented Venezuelans to access the Colombian health system amidst the COVID-19 pandemic. Migrant adults are eligible for COVID-19 vaccines, and children born to migrant parents in Colombia have access to basic health-care needs, such as childhood vaccines.

Initiatives, such as the Comprehensive Development Plan by the Economic Commission for Latin America (one of five UN regional commissions), address the structural causes of displacement by providing social and economic support, including addressing all forms of violence, strengthening asylum systems, and investing in inclusion and economic development in the countries of origin. The Global Compact on Refugees, led by UNHCR, encourages international cooperation and shared responsibility among countries of origin, transit, and destination to ensure that protection, health, and psychosocial care is provided to migrant minors, as determined in the CRC. An interdisciplinary, multilevel approach is required to ensure that migrant children and adolescents receive the mental and physical support they need to heal from their experience and feel safe wherever they live.

